# Supplementary material for: Inhibitors of the Plasmodium falciparum Hsp90 towards Selective Antimalarial Drug Design: The Past, Present and Future
Source: Cells. 2021 Oct 22;10(11):2849. doi: 10.3390/cells10112849 (PMC8616389; doi:10.3390/cells10112849)
Supplement: Supplementary file 1 [file cells-10-02849-s001.zip › cells-1413458-supplementary.pdf]

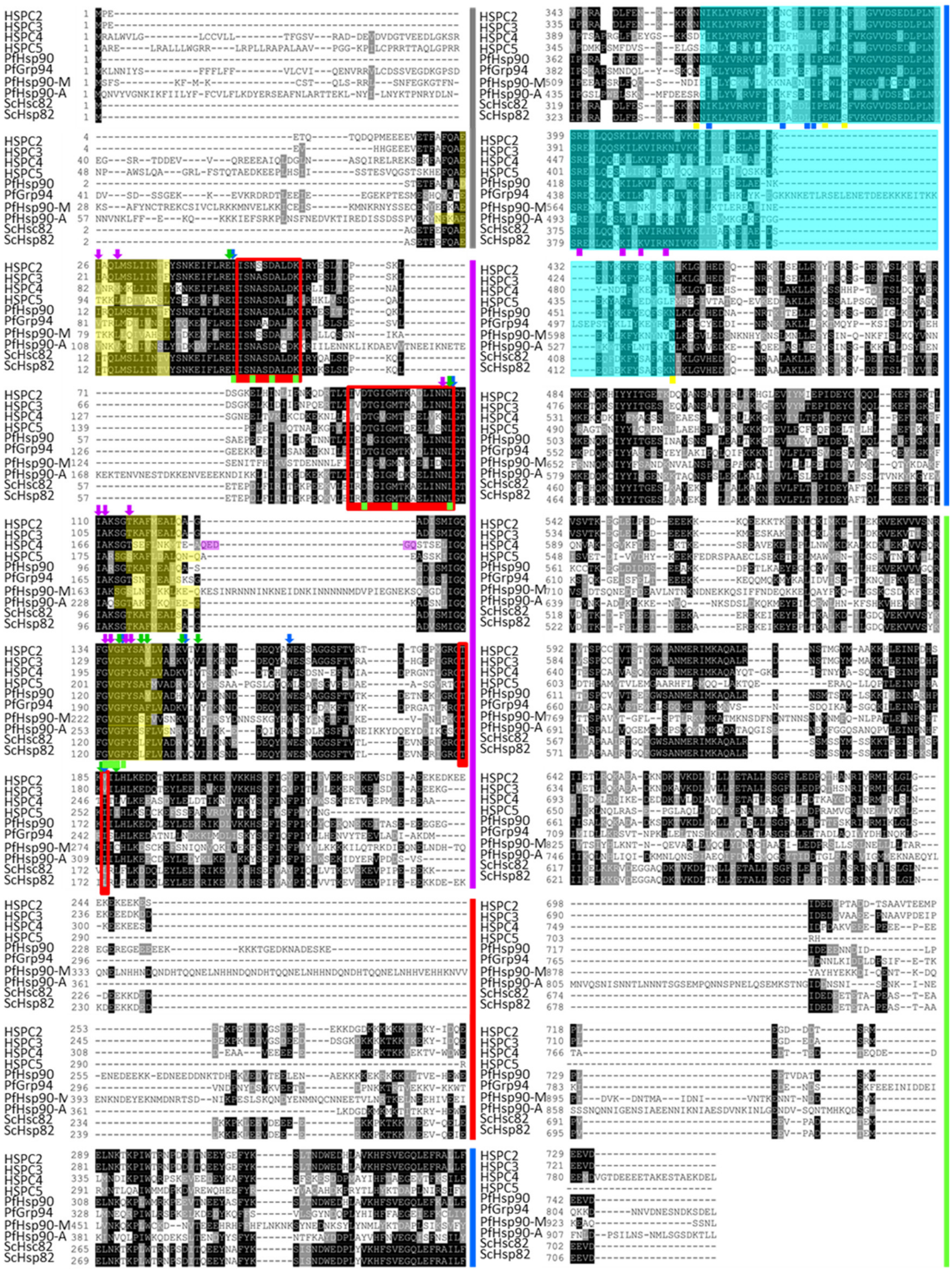

Figure S1. Multiple sequence alignments of Hsp90 proteins. Multiple sequence alignments of Hsp90 proteins from host *Homo sapiens* with sequences retrieved from NCBI (https://www.ncbi.nlm.nih.gov/protein) with respective accession number HSPC2 (NP_001017963.2), HSPC3 (NP_031381.2), HSPC4 (AAH66656.1) and HSPC5 (AAAC24722.1).The *P, falciparum* Hsp90 protein sequences were retrieved from PlasmoDB (www.plasmoDB.org) with accession numbers PfHsp90 (PF3D7_0708400); PfGrp94 (PF3D7_1222300); PfHsp90_M (Trap1, PF3D7_1118200) and PfHsp90-A (PF3D7_1443900), respectively). The model eukaryote *Saccharomyces cerevisiae* Hsp90 protein sequences were retrieved from https://www.yeastgenome.org/ ScHsc82 (accession numbers: S000004798) and ScHsp82 (Accession number: S000006161). Residues involved in ATP binding are indicated in red boxes [30,31]. The hydrophobic side pocket 1 is represented in blue, side pocket 2 in green and side pocket 3 in purple, arrow heads. The QEDGQ insertion of human Grp94 is highlighted in purple. The lid helixes N1, N4 and N5 are highlighted in yellow. The residues that take part in the

binding of geldanamycin in the NTD are indicated with green squares. The putative substrate binding pocket of the MD is shown in cyan. Critical hydrophobic residues in this cavity are indicated with blue squares. Residues involved in hydrogen bonding with GBA are indicated with yellow squares. Critical catalytic loop residues of the MD involved in ATP hydrolysis is indicated with purple squares. Colored bars to the right of the MSA indicates the protein domain: Grey is the signal peptide and Pre-N region. The NTDs are indicated with purple bars, the linker with red, the MDs with blue and the CTDs with green. The conserved residues are shown with white text in black background and semi-conserved residues are shown with white text on a grey background. The protein sequences were submitted to T-Coffee (http://tcoffee.crg.cat/apps/tcoffee/do:regular) for alignment. Boxshade (http://www.ch.embnet.org/software/BOX_form.html) conservation level colouring.
